# Biomonitoring of Non-Dioxin-Like Polychlorinated Biphenyls in Transgenic Arabidopsis Using the Mammalian Pregnane X Receptor System: A Role of Pectin in Pollutant Uptake

**DOI:** 10.1371/journal.pone.0079428

**Published:** 2013-11-13

**Authors:** Lieming Bao, Chen Gao, Miaomiao Li, Yong Chen, Weiqiang Lin, Yanjun Yang, Ning Han, Hongwu Bian, Muyuan Zhu, Junhui Wang

**Affiliations:** Department of Biotechnology, Institute of Genetics, College of Life Sciences, Zhejiang University, Zijingang Campus, Hangzhou, China; Kansas State University, United States of America

## Abstract

Polychlorinated biphenyls (PCBs) are persistent organic pollutants damaging to human health and the environment. Techniques to indicate PCB contamination *in planta* are of great interest to phytoremediation. Monitoring of dioxin-like PCBs in transgenic plants carrying the mammalian aryl hydrocarbon receptor (AHR) has been reported previously. Herein, we report the biomonitoring of non-dioxin-like PCBs (NDL-PCBs) using the mammalian pregnane X receptor (PXR). In the transgenic Arabidopsis designated NDL-PCB Reporter, the *EGFP-GUS* reporter gene was driven by a promoter containing 18 repeats of the xenobiotic response elements, while PXR and its binding partner retinoid X receptor (RXR) were coexpressed. Results showed that, in live cells, the expression of reporter gene was insensitive to endogenous lignans, carotenoids and flavonoids, but responded to all tested NDL-PCBs in a dose- and time- dependent manner. Two types of putative PCB metabolites, hydroxy- PCBs and methoxy- PCBs, displayed different activation properties. The vascular tissues seemed unable to transport NDL-PCBs, whereas mutation in *QUASIMODO1* encoding a 1,4-galacturonosyltransferase led to reduced PCB accumulation in Arabidopsis, revealing a role for pectin in the control of PCB translocation. Taken together, the reporter system may serve as a useful tool to biomonitor the uptake and metabolism of NDL-PCBs in plants.

## Introduction

Polychlorinated biphenyls (PCBs) are a class of chlorinated aromatic hydrocarbon chemicals that were used worldwide as heat-exchanger, transformer, and hydraulic fluids from the 1930s to the early 1980s [1]. When research in the 1970s indicated that these chemical additives represented a serious threat to human health and the environment, their production was gradually phased out. However, PCBs are one type of the persistent organic pollutants (POPs) that persist in the environment and bioaccumulate through the food chain [2]. Damaging effects on the immune, reproductive and endocrine systems and on tumorigenesis have been experimentally shown [3]–[7].

Different types of PCBs, known as congeners, are distinguished by the number of chlorine atoms and their location on the biphenyl ring structure. These congeners can be divided into two major groups, namely dioxin-like and non-dioxin-like PCBs [8], [9]. Dioxin-like PCBs (DL-PCBs) have one or no chlorine atoms at the *ortho*-positions and adopt a more coplanar structure. Non-dioxin-like PCBs (NDL-PCBs) have at least two chlorine substitutions at the *ortho*-positions and are less coplanar.

NDL-PCBs bind to the pregnane X receptor (PXR) in the nucleus of mammalian cells [9]–[14]. Upon interaction with a ligand from a variety of endogenous or exogenous chemicals, PXR forms a heterodimer with the retinoid X receptor (RXR) and binds to the xenobiotic response elements (XREs) located in the promoter regions of the target genes, thereby regulating their transcription. These target genes encode three phases of xenobiotic metabolizing enzymes, typically, *CYP3A4*, *UGT1A1* (UDP- glucuronosyltransferase 1A1), and *MDR1* [15], [16]. Using chromatin immunoprecipitation and next-generation sequencing technology, *in vivo* XRE signatures for PXR DNA-binding have been recently suggested [17].

Phytoremediation is an emerging technology that uses plants and associated bacteria or endophytes to facilitate environment-friendly treatment of soil, water or air contaminated by toxic pollutants [18]. Promises of phytoremediation of PCBs have been well-documented and several new trends in this field have been identified including enhancement of plant-microbe interactions [19]–[21], generation of transgenic plants expressing PCB-degrading enzymes [22]–[25], and engineering the biphenyl catabolic pathway of bacteria [26].

To evaluate the efficiency of phytoremediation, the concentrations of PCBs must be dynamically detected. Traditional detection methods for PCBs have been mostly based on GC-MS (gas chromatography-mass spectrometry) and LC-MS (liquid chromatography-mass spectrometry) analysis, which both require a series of sample preparation steps. Biomonitoring of PCBs is a novel approach that uses biological responses to assess changes in the amount of toxic chemicals [27]–[29]. This method should facilitate the evaluation of phytoremediation in real-time and on site. On the other hand, in recent years, bioavailability has been proposed to be a major step forward in understanding the environmental fate and risk of hydrophobic organic contaminants such as PCBs [30]–[32]. Monitoring of PCBs using transgenic plants carrying reporter genes offers a new route for the efficient and precise measurement of PCB bioavailability.

Biomonitoring of DL-PCBs using transgenic plants with mammalian AHR receptors has been reported [33], [34]. These transgenic plants showed inducible reporter gene expression in response to AHR ligands [33], [34]. Here, we tried to bioassay NDL-PCBs using the mammalian PXR receptor system. We found that the expression of the reporter gene was insensitive to endogenous secondary metabolites in live cells, but responded to all tested NDL-PCBs in a dose and time dependent manner. We also found that mutation in *QUASIMODO1,* encoding a 1,4-galacturonosyltransferase for pectin biosynthesis, led to reduced PCB accumulation and reporter gene expression in Arabidopsis. Taken together, this reporter system may serve as a useful tool to biomonitor the uptake and metabolism of NDL-PCBs in plants.

## Materials and Methods

### Plant Materials and Growth Conditions

*Arabidopsis thaliana* ecotype Columbia-0 was used as the source of materials for transformation. Seeds were surface-sterilized and cultured aseptically on 9 cm Petri dishes containing Gamborg’s B5 medium with 1% (w/v) sucrose and 1% (w/v) agar. The plates were maintained at 4°C for 2 d, and then transferred to a culture room (23°C, 80 µM m^−2 ^s^−1^ irradiance with a 16-h photoperiod, 30–40% RH).

### Chemicals

PCB congeners and their derivatives were supplied by AccuStandard (Connecticut, USA) from a local distributor (J&K Chemicals, Shanghai, China). PCBs and hydroxyl PCBs were dissolved in DMSO (dimethyl sulfoxide), while methoxy PCBs were dissolved in isooctane. The concentrations of organic solvents in the control or treatment media were maintained at 0.1% (v/v) or below. The following abbreviations for chemicals were used. PCB 18∶2,2′,5-Trichlorobiphenyl; PCB 28∶2,4,4′-Trichlorobiphenyl; PCB 153∶2,2′,4,4′,5,5′-Hexachlorobiphenyl; PCB 77∶3,3′,4,4′-Tetrachlorobiphenyl; PCB 126∶3,3′,4,4′,5-Pentachlorobiphenyl; H-3004∶4-Hydroxy-2,2′,5′-trichloro biphenyl; H-3005∶4-Hydroxy-2′,3,5′-trichlorobiphenyl; M-3004∶4-Methoxy-2,2′,5′-trichlorobiphenyl; M-3005∶4-Methoxy-2′,3,5′- trichlorobiphenyl; M-3006∶4-Methoxy-2′,4′,6′ -trichlorobiphenyl. Three typical PXR agonists, 4-hydroxyl-tamoxifen, RU486, and clotrimazole, were used as positive controls.

### Vector Construction and Plant Transformation

**Figure 1:**
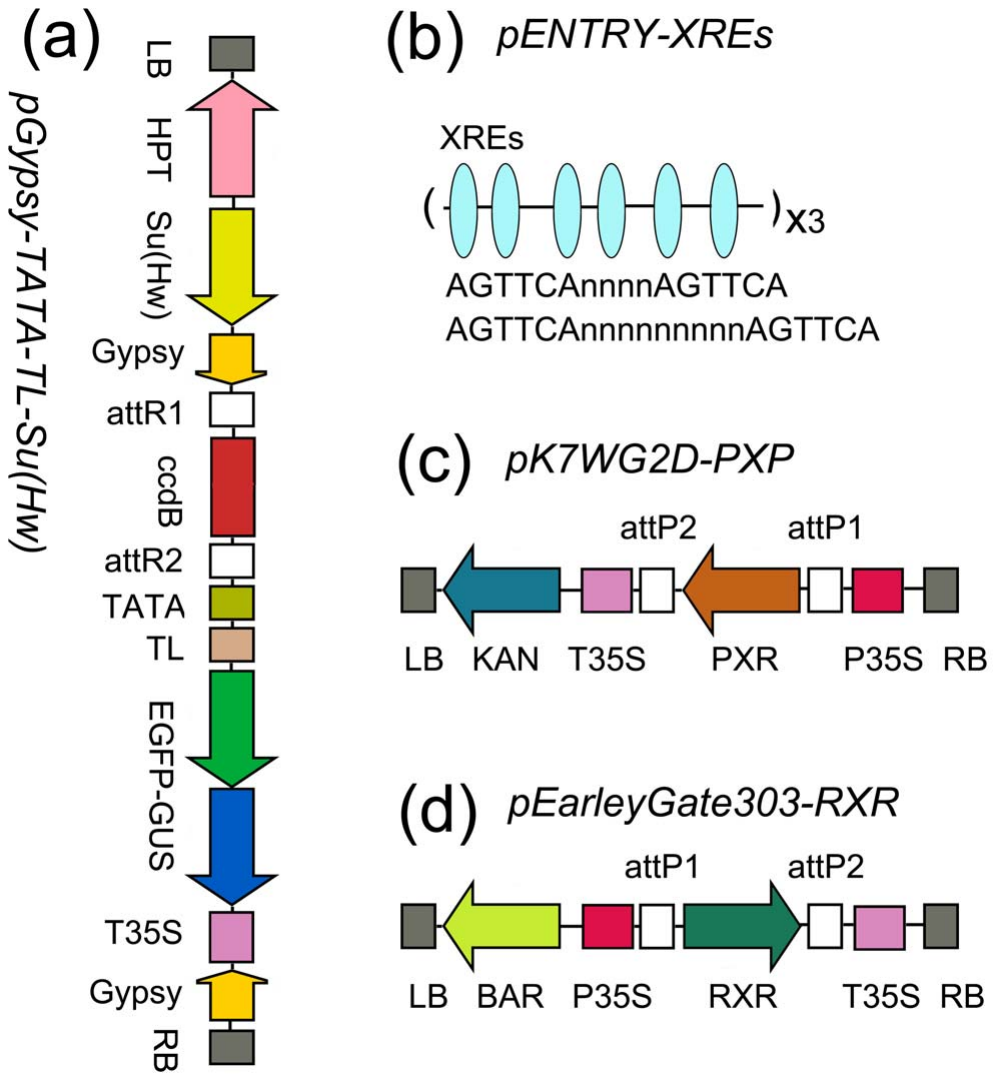
Construction of T-DNA vectors carrying the mouse PXR (pregnane X receptor) system. (a) pGypsy-TATA-TL-Su(Hw) was a destination vector for enhancer analysis which was constructed based on pGypsy-TL-Su(Hw) (She *et al.*, 2010). The positions and orientations of the selectable marker gene *HPT* (hygromycin phosphotransferase) and the reporter gene *EGFP-GUS* (Enhance Green Fluorescent Protein plus β-glucuronidase) are shown with respect to the left border (LB) and right border (RB) of the T-DNA regions. The *ccdB* region was expected to be replaced by an enhancer sequence by a LR reaction. A *35S* CaMV mini promoter (−46 to 0, TATA) together with a translation leader (TL) region from pH 7WG2D (Karimi *et al.*, 2002) were inserted upstream the reporter gene. (b) Schematic overview of the xenobiotic response elements (XREs) used in this study. The sequence was derived from the proximal region of *CYP3A11* mouse promoter and modified with the PXR DNA-binding signatures reported recently (Cui *et al.*, 2010). (c) and (d) Mouse *PXR* and *RXR* (retinoid X receptor) were co-expressed using the vectors pK7WG2D (Karimi *et al.*, 2002) and pEarleyGate303 (Earley *et al.*, 2006), respectively.

To biomonitor NDL-PCBs in transgenic Arabidopsis, we first constructed expression vectors containing three elements of the mammalian PXR system: PXR, RXR, and XREs (Fig. 1). A fused *EGFP* (Enhance Green Fluorescent Protein) and *GUS* (β-glucuronidase) gene was used as the reporter gene (Fig. 1a). This reporter gene was driven by a synthetic sequence consisting of 18 repeats of XREs (Fig. 1b). Two mouse genes *PXR* and *RXR* were overexpressed under the control of the *35S* promoter (Fig. 1c, d).

We used the GATEWAY™ system for vector construction. First, entry vectors were created using the pENTR™/D-TOPO kits (Invitrogen). PCR primers for the cloning of the coding regions of mouse *PXR* and *RXR* and the PXR DNA-binding sequence were listed in Table S1. The PXR DNA-binding sequence, which consisted of 18 repeats of xenobiotic response elements (XREs), was first created by custom DNA synthesis (Table S1). Each entry clone was confirmed by DNA sequencing. Then, the LR Reaction was conducted to generate different expression vectors. To overexpress *PXR* and *RXR* in plants, GATEWAY™ compatible destination vectors pK7WG2D [35] and pEarleyGate303 [36] were used respectively. These overexpression vectors were combined by insertion of the *PXR* expression cassette into the *HindIII* site downstream of the *35S* terminator in the pEarleyGate303-RXR vector. To counteract possible position effects and to ensure inducible expression of reporter genes, a destination vector, pGypsy-TATA-TL-Su(Hw), was constructed by insertion of a *35S* CaMV mini promoter (−46 to 0, TATA) and a translation leader (TL) region from pH7WG2D [35] into *ApaI* and *KpnI* sites of pGypsy-TL-Su(Hw) [37]. PCR primers for the construction of this destination vector were listed in the Table S1.

All of the expression vectors were electroporated into *Agrobacterium tumefaciens* strain GV3101. Plants were transformed using the vacuum infiltration method. Transgenic plants were selected on B5 plates with 12.5 µg/mL hygromycin, 25 µg/mL kanamycin, or 7.5 µg/mL phosphinothricin depending on the selection markers. To estimate the number of transgenic integration loci, about 100–150 seeds from plants of T1 generation were plated on a medium containing corresponding antibiotics. Transgenic lines whose progeny showed Mendelian characteristics (3∶1 segregation, chi-squared test) were termed as single-locus lines and chosen for further investigation [37]. The expression of mouse PXR and RXR genes in Arabidopsis was checked by RT-PCR [38].

### Histochemical Staining and Microscopy

For histochemical detection of GUS activities, young seedlings at different developmental stages and from different parts of the transgenic plants were collected [37]. They were stained at 37°C overnight in 1 mM X-Gluc (5-bromo-4-chloro-3-indolyl-b-D-glucuronic acid), 1 mM potassium ferricyanide, 0.1% Triton X-100, and 0.1 M sodium phosphate buffer, pH 7.0 with 10 mM EDTA. Samples were washed in 70% ethanol to remove chlorophyll [37]. Certain flavonoids, naringenin chalcone, kaempferol and quercetin, in fresh Arabidopsis seedlings, were stained with 0.25% (w/v) DPBA (diphenyboric acid-2-aminoethyl ester) and detected under an FITC filter [39]. The *DR5::GFP* line was used to indicate the distribution of endogenous auxin [40]. For cell wall staining, root tissues were embedded in GMA (glycol methacrylate) resin to prepare semithin sections. They were stained by FB 28 (fluorescent brightener 28) and observed under a DAPI filter. DIC (differential-interference-contrast) images or fluorescent images were visualized using a microscope (Nikon Eclipse 80i, Japan) with a DXM1200 CCD camera and EclipseNet software. Confocal microscopy (Zeiss LSM510, Germany) was used for the localization of fluorescent proteins.

### Quantitative Analysis of GUS Activity

GUS activity was measured through the detection of the cleavage of 4-methylumbelliferyl-β-D-glucuronide (MUG) into 4-methylumbelliferon (MU) by fluorometric assay and calculated as nmol 4-methylumbelliferon per minute and per mg of total soluble proteins [37]. The protein extraction solution contained 100 mM PBS, pH7.0, 10 mM EDTA, 10 mM β-mercaptoethanol, 0.1% Triton X-100, and 140 µM PMSF. The GUS reaction solution contained 1 mM MUG in the protein extraction solution. The reaction was carried out in 37°C for 20 minutes. Fluorescence was measured using a spectrofluorophotometer (RF-5301PC, Shimadzu Corporation, Japan) at 460 nm and excitation at 355 nm.

### Mutant Screen and Map-based Cloning

*Arabidopsis thaliana* ecotype Wassilewskija-2 (Ws-2) was mutagenized by EMS (ethylmethane sulfonate) using a method described by Anderson and Wilson [41]. A mutant, designated *pcb1*, resistant to the bleach effect of 10 µM PCB 18 was screened out from a pool of M2 generation seeds. Since *pcb1* was infertile, we picked out a heterozygote sibling from the same pool. The mutant was crossed with wild-type Col-0 to create a mapping population. Mapping of *pcb1* was performed according to the protocols of Lukowitz *et al.* [42] and Jander *et al.* [43].

### Cell Wall Analysis and PCB Measurement

Cell walls were prepared from 14-d-old seedlings by boiling in ethanol. They were then air-dried and weighed. After sulfuric acid degradation of the cell walls, monosaccharide composition and galacturonic acid content were determined by HPLC and used to calculate the percentage cell wall dry weight [44]. PCB 18 in 14-d-old seedlings was extracted and measured by a protocol described by Li *et al.* and expressed as µg PCB kg^-1^ dry weight [45].

### Statistics and Image Processing

Each treatment contained 30–50 seedlings and was replicated at least 3 times. Statistical analysis of the data was performed using Microsoft Excel and Student’s *t*-test. Images were processed using Adobe Photoshop CS2.

## Results and Discussion

### Generation of Transgenic Arabidopsis Carrying the Mouse PXR System

Arabidopsis plants were co-transformed with the *EGFP-GUS* reporter gene construction (Fig. 1a) and the *PXR/RXR* receptor and binding partner construction (Fig. 1c, d). In total, seventeen transgenic lines, termed as NDL-PCB Reporters, homozygous for both constructions, were generated. The expression of mouse *PXR* and *RXR* genes in the transgenic lines used for further studies was confirmed by the RT-PCR analysis (Fig. S1).

The mechanism for biomonitoring of NDL-PCBs in transgenic Arabidopsis was schematically depicted in Fig. S2. After binding of NDL-PCBs to the PXR receptors, the expression of reporter gene was activated. The EGFP signal could be viewed by confocal microscopy, while the GUS enzyme could transform the colorless substrate X-Gluc into blue product (histochemical assay, stain tissue blue, Fig. S2) or the non-fluorescent substrate MUG into fluorescent product (quantitative assay by fluorophotometry, Fig. S2).

### NDL-PCB Reporters were Insensitive to Endogenous Secondary Metabolites in Live Cells

**Figure 2:**
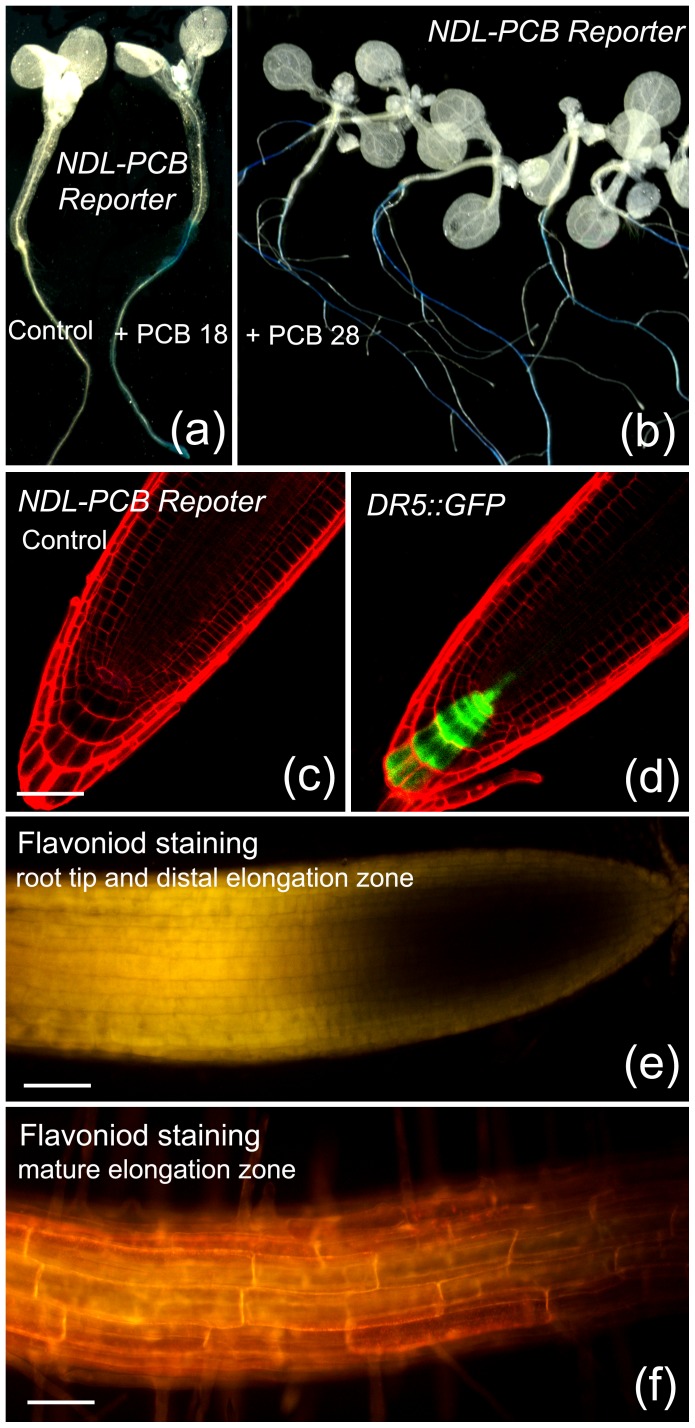
The expression of the reporter gene in NDL-PCB (non-dioxin-like polychlorinated biphenyl) Reporters was insensitive to endogenous secondary metabolites in living cells. (a) Histochemical assay of GUS activity in young seedlings of NDL-PCB Reporters. Three-day-old seedlings were transferred to plates without (control) or with 50 nM PCB 18 (+ PCB 18) and grown for 3 days. The right root under PCB 18 treatment was stained blue. (b) Histochemical assay of GUS activity in 12-day-old NDL-PCB Reporters. Six-day-old seedlings were transferred to plates with 50 nM PCB 28 and grown for 6 days. Roots under PCB 28 treatment were stained blue. (c) NDL-PCB Reporters were insensitive to endogenous auxin in root tips as no GFP signals were observed by confocal microscopy. The red PI (propidium iodide) indicated the cell wall. (d) The DR5::GFP line reported auxin peaks (GFP signals) in roots. (e) Naringenin chalcone (yellow fluorescence) in distal elongation zones and meristems of 10-d-old Arabidopsis roots. Meristematic cells were small in size. (f) Kaempferol and quercetin (gold fluorescence) in mature elongation zones of 10-day-old Arabidopsis roots. Bars for (c) to (f), 30 µm.

It has been reported that several plant secondary metabolites of dietary origin such as lignans, carotenoids and flavoniods were weak agonists of the PXR receptor in mammals [13]. We investigated whether the expression of reporter gene in NDL-PCB Reporters was responsive to endogenous metabolites. In histochemical assays, we found that young Arabidopsis seedlings of NDL-PCB Reporters displayed no background GUS activities on the medium without NDL-PCBs (Fig. 2a, c), indicating that the expression of the reporter gene was insensitive to endogenous PXR ligands. Two NDL-PCB congeners PCB 18 and PCB 28 induced GUS activities in the root parts of Arabidopsis seedlings, since the roots were stained blue, while the cotyledons and hypocotyls were not (Fig. 2a, b).

Auxin is another type of secondary plant metabolite. It has been reported that the AHR receptor-mediated reporter gene expression is responsive to plant auxin [34]. Here, we found that the expression of the reporter gene in NDL-PCB Reporters were insensitive to endogenous auxin (Fig. 2c), as compared with the DR5::GFP line, which reported auxin peaks in roots (Fig. 2d).

In living plant cells, lignans and carotenoids were deposited in the cell wall and plastid, respectively, while flavonoids were reported to localize to the plasma membrane, endomembrane system, and the nuclear region [39]. In roots of NDL-PCB Reporters, we detected the accumulation of naringenin chalcone (yellow fluorescence) in distal elongation zones and kaempferol and quercetin (gold fluorescence) in mature elongation zones (Fig. 2e, f). However, these flavonoids were mainly localized in the membrane systems, and the amount of flavonoids in the nucleus might be below the limit to activate the PXR receptor. Taken together, it seemed likely that endogenous PXR agonists were compartmentalized in live plant cells, rendering them inaccessible to the PXR receptor in the nucleus. Alternatively, only a limited amount of secondary metabolites entered into the nucleus, making them unable to drive the expression of the reporter gene.

### Dose- and Time-dependent GUS Activities in the NDL-PCB Reporter in Response to PXR Ligands

**Figure 3:**
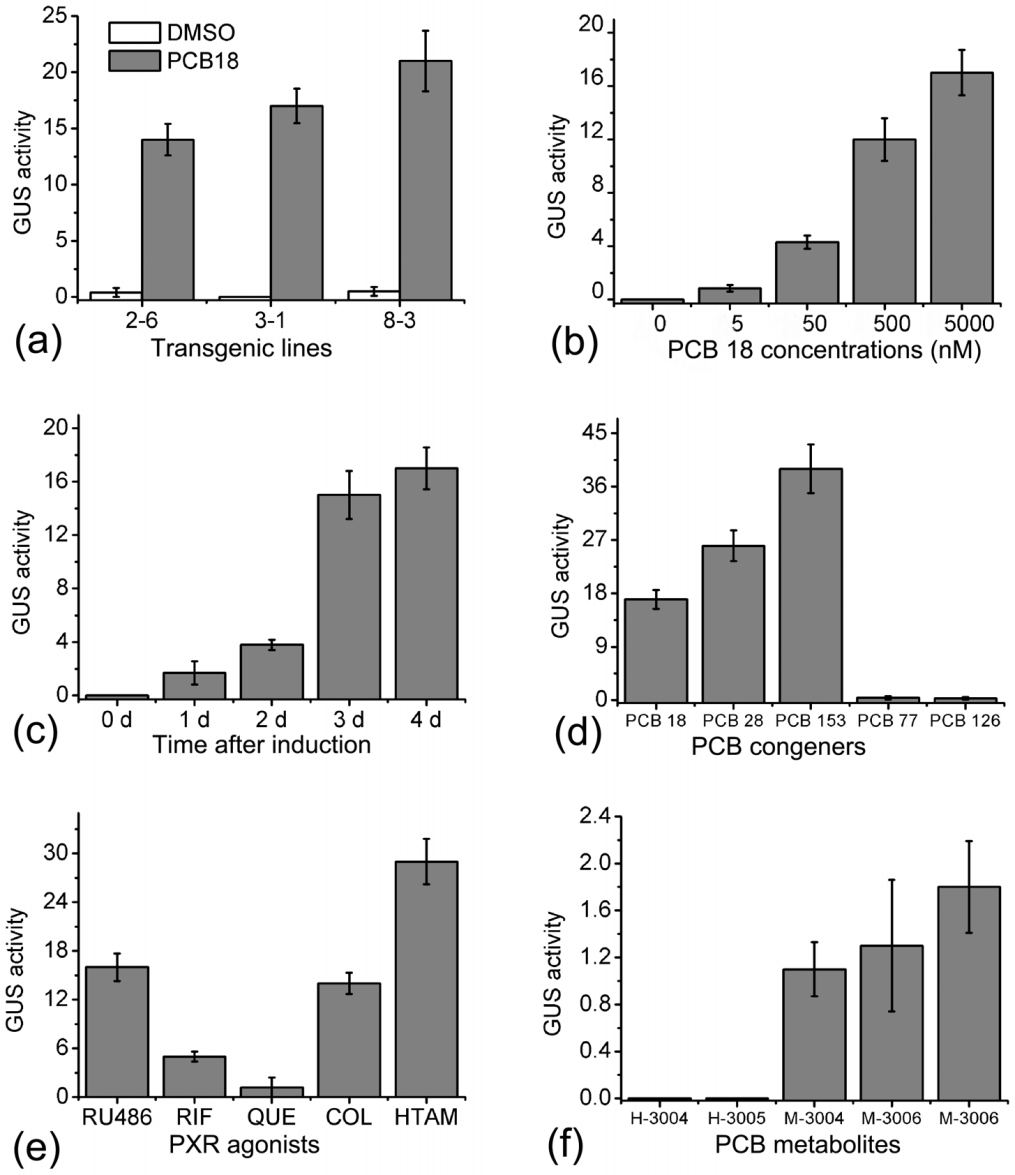
Dose- and time-dependent GUS activities in NDL-PCB Reporters in response to PXR ligands. (a) Induced GUS activity in three transgenic lines in response to 5 µM PCB 18. (b) Dose-dependent GUS activity upon treatment with various concentrations of PCB 18. (c) Time-dependent GUS activity in response to 5 µM PCB 18. (d) GUS activity was responsive to NDL-PCBs but insensitive to DL-PCBs. (e) Response of GUS activity to typical PXR agonists. (f) Methoxyl- PCBs could induce low GUS activity, while hydroxyl-PCBs could not. Six-d-old seedlings were transferred to plates with indicated chemicals and grown for 4 d before GUS activity analysis. Chemicals in (d) to (f) were at concentrations of 10 µM.

We next quantitatively assayed the inducible GUS activities in response to NDL-PCBs and other PXR ligands. All transgenic lines exhibited similar responses to PCB 18 and PCB 28, although they varied in the intensities of their GUS activities (Fig. 3a). Line 3-1 showed the lowest background and had an approximately 80- fold induction level (Fig. 3a). Thus, this line was selected for further analyses on account of its tight and strong response to NDL-PCBs. The induced GUS activity was detectable at a concentration of 5 nM and increased in a dose-dependent manner upon treatment with PCB 18 for 4 d (Fig. 3b). GUS activity also increased in response to PCB 18 in a time-dependent manner, and reached a plateau 3–4 d after treatment (Fig. 3c). Among the three NDL-PCBs, PCB 153 displayed the highest GUS activity; whereas two DL-PCBs, namely PCB 77 and PCB 126, were unable to activate the PXR receptor (Fig. 3d). Three drugs classified as typical PXR agonists, 4-hydroxyl-tamoxifen, RU486, and clotrimazole, induced GUS activity efficiently (Fig. 3e). Exogenous quercetin at 10 µM only weakly induced GUS activity (Fig. 3e). It has been reported that, in *in vitro* assays, rifampicin only activates human PXR but not mouse PXR [14], however we found that the NDL-PCB Reporter was sensitive to rifampicin (Fig. 3e). We finally tested whether or not the expression of the reporter gene was responsive to PCB metabolites. PCBs in plant cells have been reported to be hydroxylated and then methoxylated [46], [47]. Results showed that hydroxyl- PCBs were unable to activate the PXR receptor, while methoxyl- PCBs could induce low GUS activity at high concentrations (Fig. 3f).

Mammalian cultured cells transfected with reporter plasmids responsive to the PXR receptor have served as rapid assays for the effects of NDL-PCBs [9], [10], [13], [30]. Here, we implemented the mouse PXR receptor system to model plant Arabidopsis. We found that the expression of reporter gene in NDL-PCB Reporters was sensitive to NDL-PCBs and PXR agonists but not to DL-PCBs. However, we only tested a limited number of PCB congeners, further work to test more PCB congeners should help to determine the specificity and efficiency of this bioassay system.

### The Vascular Tissues Seemed Unable to Actively Transport NDL-PCBs

**Figure 4:**
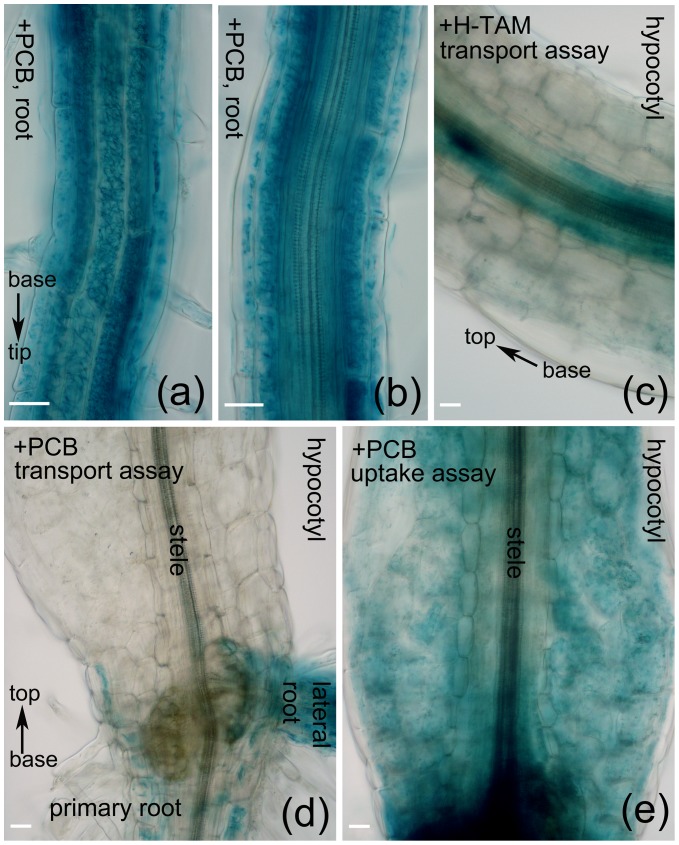
The vascular tissues of Arabidopsis seemed unable to transport NDL-PCBs actively. (a) Histochemical assay of GUS activity in root epidermal and cortical cells. (b) Histochemical assay of GUS activity in root vascular tissues. (c) Under transport assay, H-TAM (4-hydroxyl-tamoxifen) was able to induce GUS activity in the vascular tissues of hypocotyls. (d) PCB 18 was unable to induce GUS activity in the vascular tissues of hypocotyls in the transport assay. (e) Under an uptake assay, the vascular tissues of hypocotyls displayed very weak GUS activity. Six-d-old seedlings were transferred to plates with indicated chemicals and grown for 4 d before histochemical assay. Uptake assay: plates were placed vertically (90°), thus, some parts of cotyledons and hypocotyls were in contact with the media. Transport assay: plates were placed at 100°, thus, only the roots were in contact with the media. Bars, 30 µm. The orientations of roots and hypocotyls were indicated.

Since PCBs were toxic and volatile, we wrapped the Petri dishes with parafilm. When the plates were placed vertically (at 90°) on the culture racks, some parts of the cotyledons and hypocotyls of the Arabidopsis seedlings were in contact with media. By contrast, when the plates were placed at 100°, only the roots were in contact with media because of the gravitropism of the hypocotyls. We termed these two culture conditions as ‘uptake assay’ and ‘transport assay’ respectively. Under the transport assay, as PCB concentrations were higher than 5 µM, the cotyledon parts of Arabidopsis seedlings had GUS activity, but the hypocotyl parts did not, indicating that cotyledons could take up volatile PCBs originated from the medium (Fig. S3). Microscopy of histochemical stained seedlings revealed that in Arabidopsis roots, GUS activity was strong in epidermal and cortical cells but weak in the vascular tissues (Fig. 4a, b). When the hypocotyls were not in contact with medium, their vascular tissues failed to display any GUS activity (Fig. 4d, transport assay). In contrast, when the hypocotyls were in contact with medium, the epidermal and cortical cells showed strong GUS activity but their steles displayed no or very weak GUS activity (Fig. 4e, uptake assay). On the contrary, 4-hydroxyl tamoxifen, a selective estrogen receptor modulator, appeared to be transported through the vascular bundles of hypocotyls (Fig. 4c). Taken together, it seemed that in young Arabidopsis seedlings NDL-PCBs were not actively transported from roots to shoots.

The routes of PCB uptake and translocation in plants remain controversial. It has been suggested that hydrophobic PCBs enter plant leaves principally through emission from the soil rather than translocation from root to shoot [45], [48], [49]. However, several studies have demonstrated that vascular tissues can transport PCBs from root to shoot [50], [51]. The NDL-PCB Reporters generated here gave us a good opportunity to probe the pathways of PCB transport. We found that in young Arabidopsis seedlings the vascular tissues were unable to transport NDL-PCBs from roots to shoots. Since we only tested 3 NDL-PCBs in Arabidopsis, we could not rule out the possibility that a remarkable diversity in uptake and transportation of PCBs exists dependent on the specific properties of each congener and the characteristics of the plant species concerned [52].

### Mutation in *QUASIMODO1* Led to Reduced PCB Accumulation in Arabidopsis

**Figure 5:**
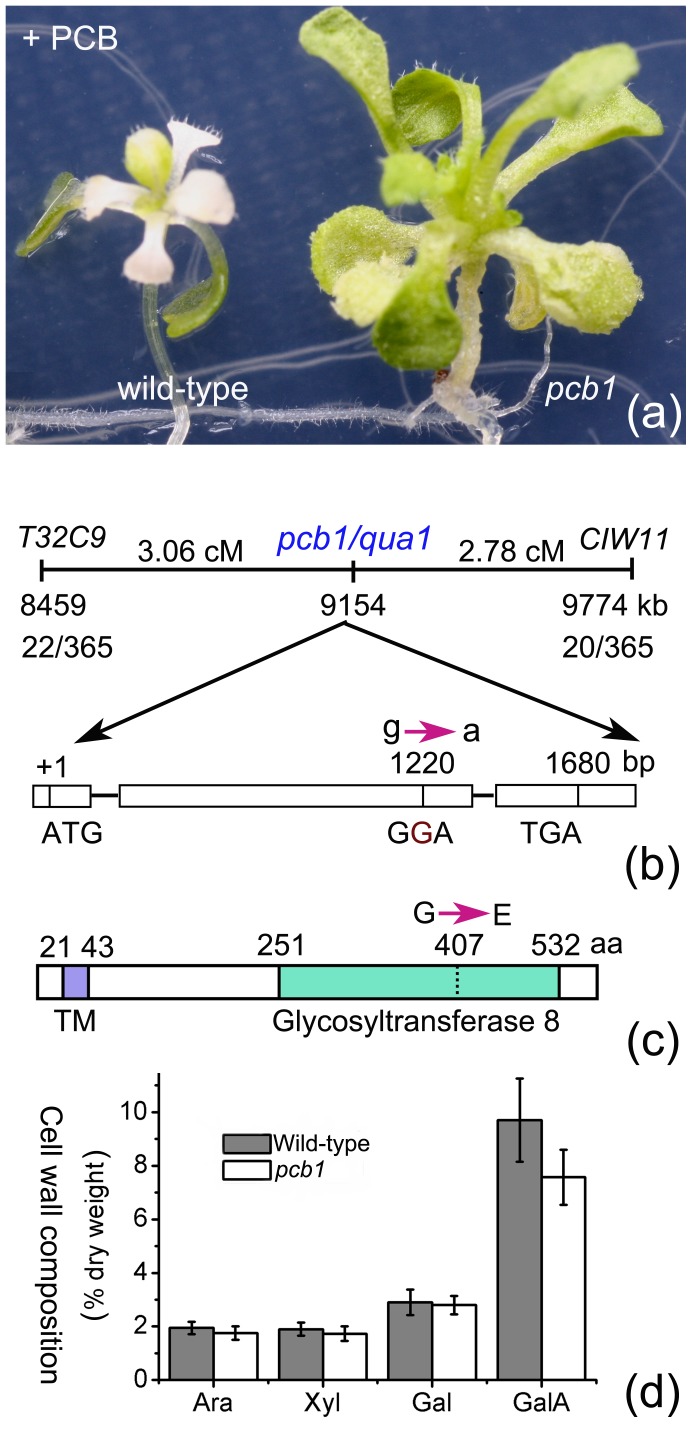
Mutation in a gene required for pectin synthesis led to PCB resistance. (a) A screened *pcb1* mutant resistant to the bleach effects of 10 µM PCB 18. Six-d-old Arabidopsis seedlings were transferred to plates containing 10 µM PCB 18 and grown for 2 weeks. (b) Map-based cloning of *pcb1* revealed it was a new allele of *quasimodo1* and had a G- to A- transition in its second exon. The positions of each locus in chromosome 3 of Arabidopsis and the recombinants in relation to each marker are shown. (c) The PCB1/QUA1 protein had a transmenbrane (TM) domain in the N-terminus and a glycosyltransferase 8 (GT8) domain in the C-terminus, while *pcb1* had a mutation in the conserved GT8 domain. (d) The arabinose (Ara), xylose (Xyl), and galactose (Gal) contents in 12-d-old seedlings of *pcb1* were comparable to the wild-type, but the GalA content showed a ∼20% reduction.

When Arabidopsis seedlings were grown in 10 µM PCB 18 for more than 4 d, newly formed leaves were bleached [53] (Fig. 5a). We screened out a mutant resistant to this bleach effect and designated it *pcb1* (Fig. 5a). Detailed phenotypical comparison between *pcb1* and wild-type was showed in Fig. S4. Positional cloning of *pcb1* was performed. The gene was mapped into a region between a CAPS marker T32C9 and a SSLP marker CIW11 on chromosome 3 (Fig. 1b). In 365 mutants of the F2 generation, 22 and 20 recombinants were identified in relation to T32C9 and CIW11 respectively, indicating that the gene was localized around the middle region between these markers (Fig. 1b). Further work revealed that *pcb1* was a new allele of *quasimodo1* which encoded a putative membrane-bound and Golgi-localized galacturonosyltransferase required for pectin synthesis [44], [54]. Sequencing of *pcb1* gene showed that it had a G- to A- transition at position 1220 in its second exon, resulting in a glycine (G) to glutamic acid (E) mutation in the conserved glycosyltransferase domain (Fig. 5b, c). We measured the monosaccharide composition and galacturonic acid (GalA) content in cell walls of wild-type and *pcb1*. Results showed that the arabinose (Ara), xylose (Xyl), and galactose (Gal) contents of *pcb1* were comparable to the wild-type, but the GalA content showed a ∼20% reduction in *pcb1*, indicating a defectiveness in pectin biosynthesis in this mutant (Fig. 5d).

**Figure 6:**
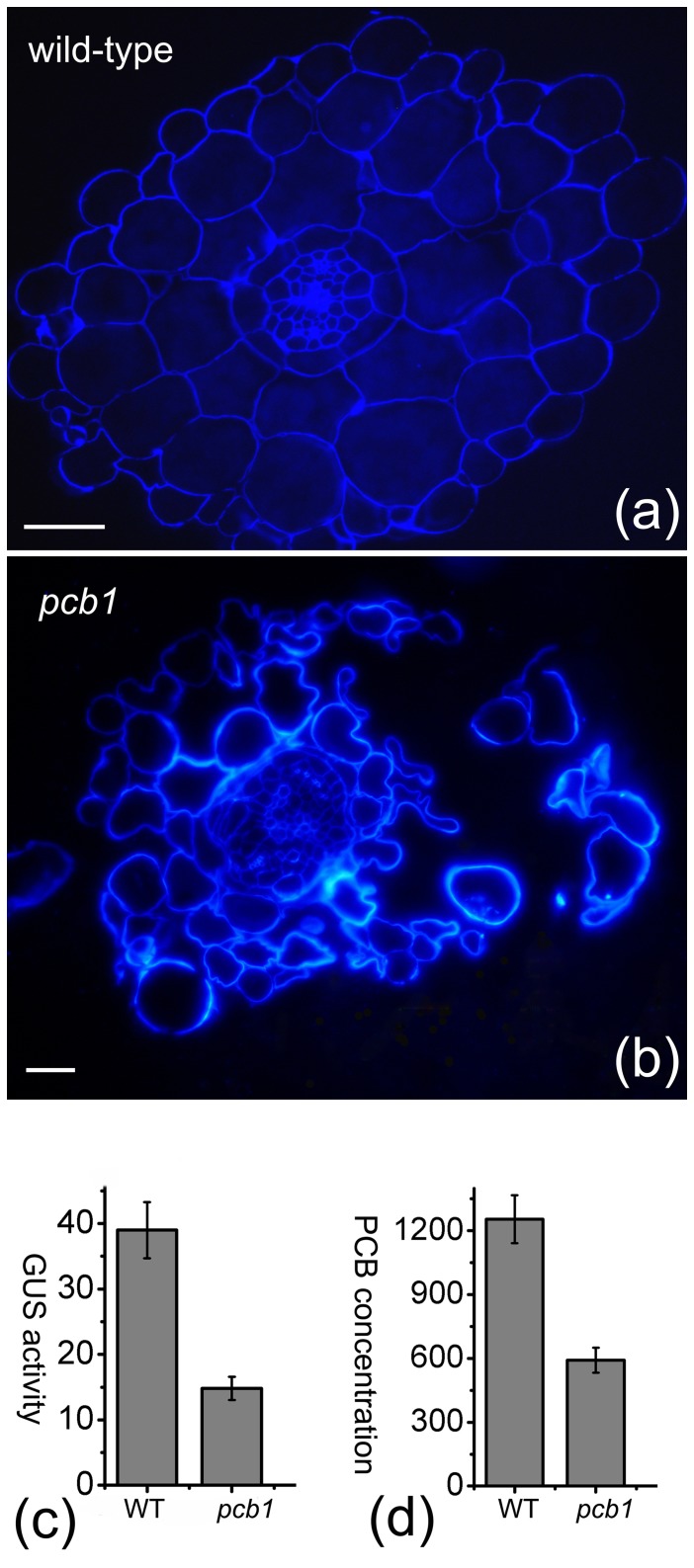
Reduced cell adhesion and PCB accumulation in the *pcb1* mutant. (a) Cell wall staining in a transverse section of a wild-type root. (b) Cell wall staining in a transverse section of a *pcb1* root showed dramatically reduced cell adhesion in epidermal, cortical and endodermal cells of roots. (c) GUS activity in the wild-type and the *pcb1* mutant induced by PCB 18. (d) Comparison of PCB 18 accumulation in wild-type and *pcb1*. Six-d-old Arabidopsis seedlings were transferred to plates containing 10 µM PCB 18 and grown for 6 d. Bars: 12.5 µm.

The *pcb1* mutant showed dramatically reduced cell adhesion in epidermal, cortical and endodermal cells of roots, but the vascular tissues of roots seemed less affected (Fig. 6a). We crossed *pcb1* with NDL-PCB Reporter and isolated a line carrying both the mutation and the reporter system. PCB 18 accumulation and GUS activity in the *pcb1* line and the wild-type of 14-d-old seedlings were then measured. Results showed that the mutant had a 62% reduction in GUS activity (Fig. 6c) and a 53% reduction in PCB content (Fig. 6d), indicating agreement between the bioassay and the traditional detection method.

Pectins are plant cell wall polysaccharides based on α-1,4-linked D-galacturonic acids. The properties of pectins depend on the degree of cross-linking, methylated and acetylation [55], [56]. The role of pectin in chelation of heavy metal ions has been well-documented [57], [58], but the possible connection between pectin and the volatile environmental pollutants remain unclear. It has been reported that airborne PCBs may be captured by two pathways, the cuticle and the stomata [59]. Here, we found that mutation in *PCB1*/*QUA1* encoding a 1,4-galacturonosyltransferase led to reduced pectin biosynthesis and PCB accumulation. Pectins are synthesized in the Golgi apparatus by QUA1 and subsequently by QUA2, a pectin methyltransferase, in a highly methyl-esterified form [60]. Perhaps the reduced cell adhesion in *pcb1* mutant blocked the uptake and translocation of PCBs physically; and/or, the altered chemical properties of cell walls in *pcb1* mutant retarded the translocation of PCBs. It is also possible that the changed secondary metabolism of carbohydrates in *pcb1* boosted detoxification or deactivation reactions in Arabidopsis cells. Further work is required to address these questions.

## Conclusion

We developed an efficient system for biomonitoring of NDL-PCBs in transgenic Arabidopsis. With the help of this system, we found that the vascular tissues could not transport NDL-PCBs efficiently, and pectins were required for the uptake and translocations of NDL-PCBs. The system should not only help to evaluate the bioavailability and phytoremediation of NDL-PCBs, but also help to address their cellular localization and metabolism in plant cells. Once the reporter gene is replaced with a functional gene, it may improve the efficiency of phytoremediation.

## Supporting Information

Figure S1 **RT-PCR analysis of the expression of two mouse genes** ***PXR*** **and** ***RXR*** **in transgenic Arabidopsis.**
(JPG)

Figure S2 **A schematic depiction of the mechanism for biomonitoring of NDL-PCBs in transgenic Arabidopsis.**
(JPG)

Figure S3 **Cotyledons could take up volatile PCBs from the medium.**
(JPG)

Figure S4 **Detailed phenotypical comparison between** ***pcb1*** **and wild-type.**
(JPG)

Table S1 **PCR primer sequences used in this study.**
(PDF)
