# Supplementary figures and images for: Biomonitoring of Non-Dioxin-Like Polychlorinated Biphenyls in Transgenic Arabidopsis Using the Mammalian Pregnane X Receptor System: A Role of Pectin in Pollutant Uptake
Source: PLoS One. 2013 Nov 13;8(11):e79428. doi: 10.1371/journal.pone.0079428 (PMC3827382; doi:10.1371/journal.pone.0079428)

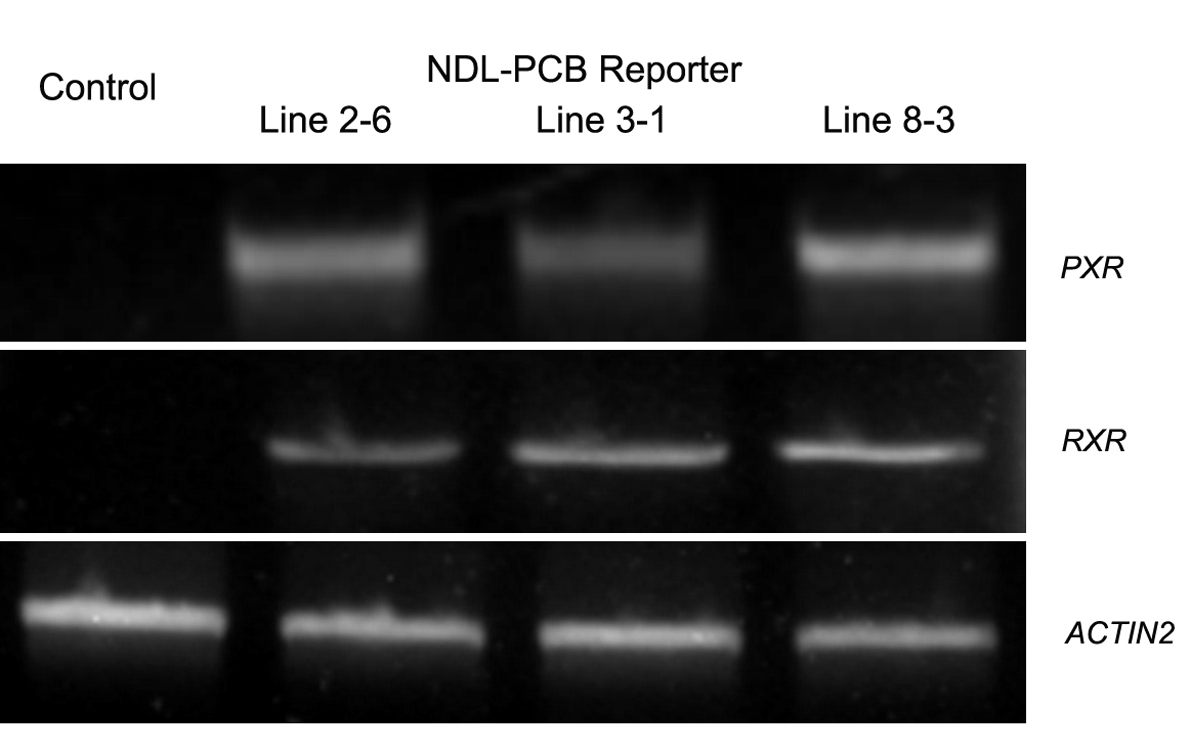

Supplement: Figure S1 — RT-PCR analysis of the expression of two mouse genes PXR and RXR in transgenic Arabidopsis. (JPG) [file pone.0079428.s001.jpg]

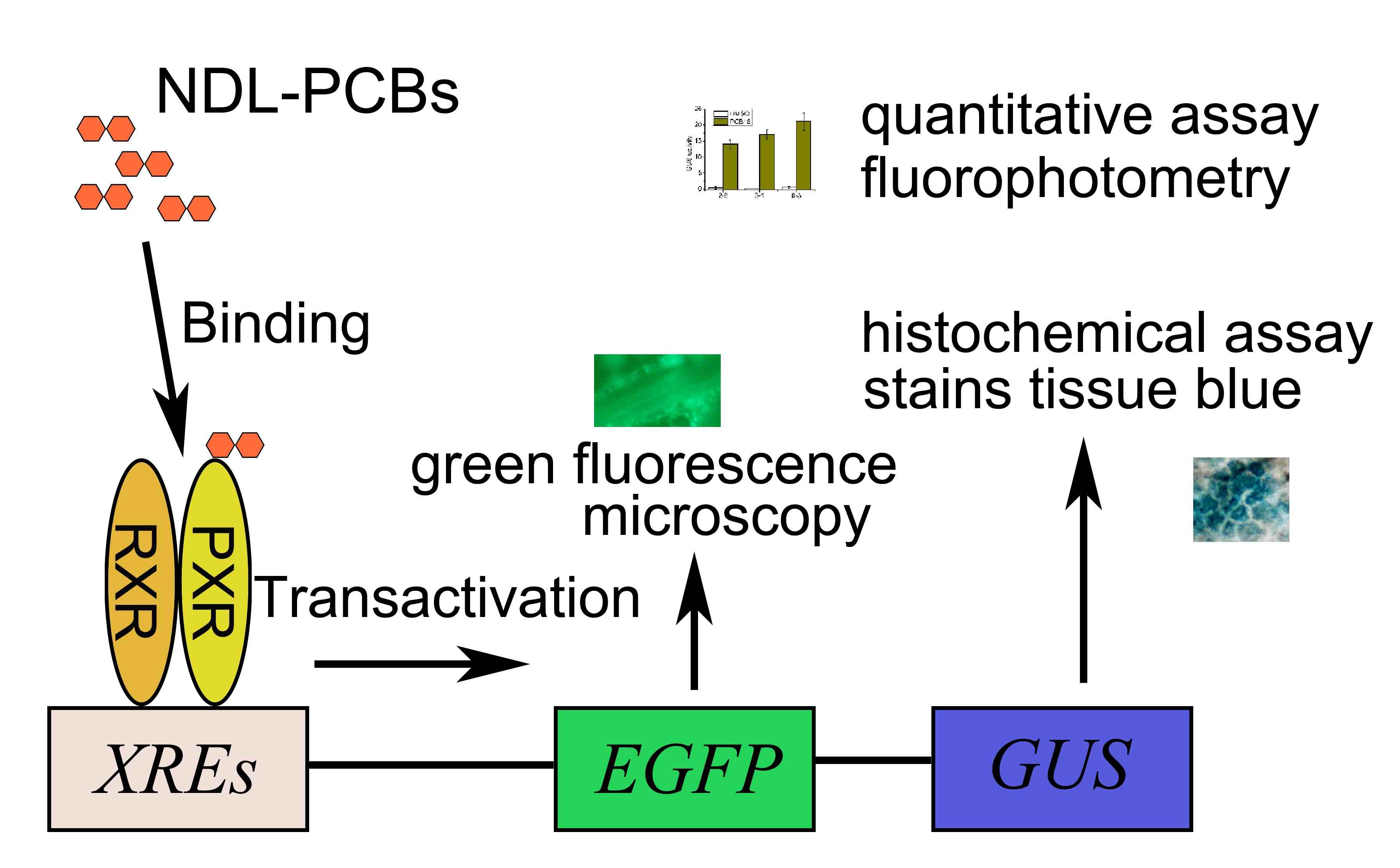

Supplement: Figure S2 — A schematic depiction of the mechanism for biomonitoring of NDL-PCBs in transgenic Arabidopsis. (JPG) [file pone.0079428.s002.jpg]

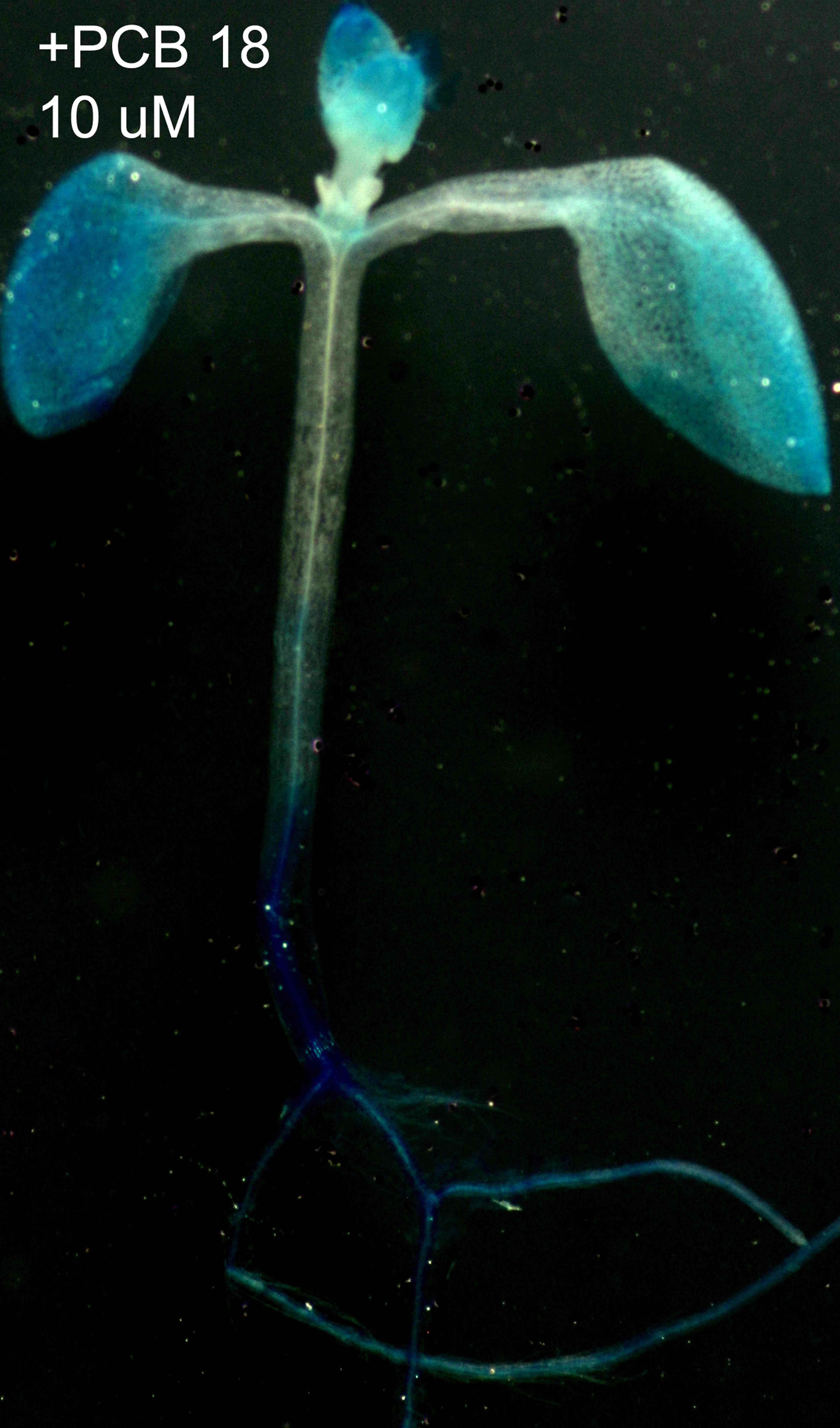

Supplement: Figure S3 — Cotyledons could take up volatile PCBs from the medium. (JPG) [file pone.0079428.s003.jpg]

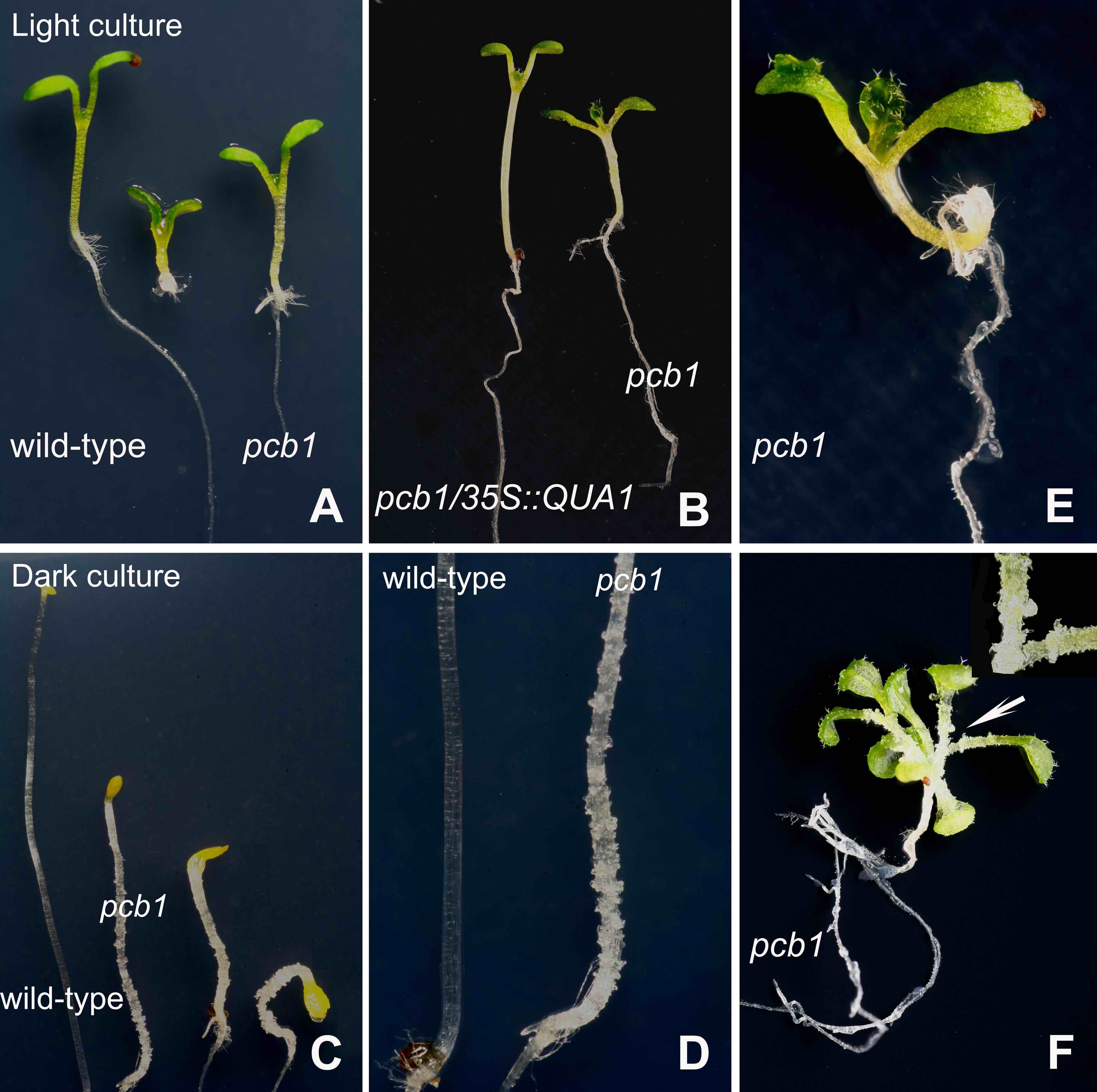

Supplement: Figure S4 — Detailed phenotypical comparison between pcb1 and wild-type. (JPG) [file pone.0079428.s004.jpg]
