# Supplementary material for: Biomonitoring of Non-Dioxin-Like Polychlorinated Biphenyls in Transgenic Arabidopsis Using the Mammalian Pregnane X Receptor System: A Role of Pectin in Pollutant Uptake
Source: PLoS One. 2013 Nov 13;8(11):e79428. doi: 10.1371/journal.pone.0079428 (PMC3827382; doi:10.1371/journal.pone.0079428)
Supplement: Table S1 — PCR primer sequences used in this study. (PDF) [file pone.0079428.s005.pdf]

Table S1. Primer sequences used in this study

Primers	Primer Sequence 5'-3'
generating entry clone for coding sequence	
PXR-Up	CACCATGAGACCTGAGGAGAGCTG
PXR-Dn	GCCATCTGTGCTGCTAAATA
RXR-Up	CACC ATGGACACCAAACATTTCCT
RXR-Dn	GGTGGCTTGATGTGTGCCT
generating entry clone for XREs	
XRE-Up	CACCATGTTAGCTCAAAGTAGGTCA
XRE-Dn	AAGCCAGTAGATGGATCACCT
generating destination clone for enhancer analysis	
TATA-TL-Up	TTAC GGGCCC ACTAGTGCGGCCGCCT *ApaI*
TATA-TL-Dn	AACT GGTACC CGCAAGACCCTTCCTCTATAT *KpnI*

XREs:

ATGTTAGCTCAAAGTAGGTCAAGTTGGGCAGAGTACCAAAGTCCAGTGATGCAAAGGTGATCCATCTACTGGCTTGATCCATGTTAGCTCAAAGTAGGTCAAGTTGGGCAGAGTACCAAAGTCCAGTGATGCAAAGGTGATCCATCTACTGGCTTGATCCATGTTAGCTCAAAGTAGGTCAAGTTGGGCAGAGTACCAAAGTCCAGTGATGCAAAGGTGATCCATCTACTGGCTT
